# Vasospastic individuals demonstrate significant similarity to glaucoma patients as revealed by gene expression profiling in circulating leukocytes

**Published:** 2009-11-14

**Authors:** Kristina Yeghiazaryan, Josef Flammer, Selim Orgül, Kerstin Wunderlich, Olga Golubnitschaja

**Affiliations:** 1 Department of Radiology, University of Bonn, Germany; 2 University Eye Clinic, Basel, Switzerland

## Abstract

**Purpose:**

There is growing evidence that vasospatic individuals could be predisposed to develop glaucoma. Vasospastic deregulation is ensuing in activation of circulating leukocytes. In previous studies using “gene-hunting” strategies, we demonstrated stable alterations in gene expression profiles of circulating leukocytes isolated from glaucoma patients with vascular deregulation when compared to healthy individuals with no history of glaucomatous damage. The goal of this study was to look for possible similarities in gene expression profiles of circulating leukocytes in vasospastic individuals and glaucoma patients.

**Methods:**

Normal-tension (NTG) and high-tension (HTG) glaucoma patients as well as individuals with vascular deregulation (VD) and healthy controls were recruited for the gene expression analysis. The methodology of comparative Expression Array analysis followed by highly sensitive quantitative real-time PCR has been used.

**Results:**

Compared to the control group the expression of 146, 68, and 60 genes was found to be altered in NTG, HTG, and VD groups respectively. Thirty-four genes demonstrated similar expressional alterations in NTG, HTG, and VD groups versus controls, and only 21 genes demonstrated similar expressional alterations in NTG and HTG groups, having no overlap with the VD group.

**Conclusions:**

This result indicates a potential predisposition of vasospastic individuals to glaucomatous optic nerve atrophy. The targeted expression profiles might be further considered for early/predictive glaucoma diagnosis.

## Introduction

Glaucomatous optic neuropathy (GON) is characterized by a combination of retinal ganglion cell loss, activation of astrocytes, and remodeling of the lamina cribrosa. The etiology of glaucoma and risk factors are only partially known [1,2]. Although elevated intraocular pressure (IOP) has been shown to be the major risk factor, there is a cohort of patients, sometimes even at younger ages with normal IOP, developing normal-tension glaucoma (NTG). A wealth of literature points to the potential importance of hemodynamics in NTG, but randomized controlled trials are not yet available [3]. A valuable diagnostic tool for ascertaining vasospastic diathesis is the nailfold capillary microscopy. The best known blood-born factor is an increased plasma level of endothelin-1 to estimate vasospastic diathesis [4]. Vasospasm, frequently observed in the young female subpopulation can potentially predispose to several disorders, including glaucomatous optic nerve head atrophy. Ocular ischemia resulting from blood flow deficits may play a major role in the initiation of glaucoma. Indeed, hypoxia followed by high secretion of excitatory amino acids and elevated levels of intracellular calcium may eventually lead to retinal ganglion cells death [3,5,6]. The molecular pathways involved in vasospastic deregulation, which can potentially impact the GON, have not yet been investigated. In our previous studies, we demonstrated stable alterations in gene expression of circulating leukocytes isolated from glaucoma patients compared to controls [7–10]. The goals of this study were

- identification of possible similarities as well as dissimilarities in gene expression profiles of circulating leukocytes between vasospastic individuals and glaucoma patients;
- identification of specific gene transcription patterns in circulating leukocytes of glaucoma patients;
- selection of potential molecular targets in blood for noninvasive early diagnostics of different glaucoma forms.

## Methods

### Recruitment of patients and controls

Altogether 79 patients and controls were recruited at the University Eye Clinic in Basel. Patients and controls were well matched together from viewpoint of age and gender. Blood samples (20 ml) were collected from 42 non-glaucomatous non-vasospastic individuals (controls, group 1), six non-glaucomatous vasospastic individuals (VD), ten normal-tension glaucoma (NTG) individuals, ten high-tension glaucoma (HTG) individuals, and 11 pseudoexfoliation glaucoma (PEX) individuals. All glaucoma patients had bilateral typical glaucomatous optic nerve head cupping and visual field defects with a mean deviation greater than 7 dB in the Octopus program G1. For NTG patients, IOP never exceeded 21 mm Hg, as assessed in at least two diurnal tension curves. The diagnosis of vasospasm was based on nailfold capillaromicroscopy findings. After local cooling of a finger, all vasospastic individuals exhibited a stop in blood flow for more than 20 s. Controls had an unremarkable ophthalmologic examination and did not show any vasospastic response. No patient or control subject had received either systemic or local ocular therapy at least 4 weeks before the study. All investigations conformed with the principles outlined in the Declaration of Helsinki and were performed with permission from the Ethic’s Committee of the Medical Faculty, University of Basel, Switzerland (ClinicalTrials.govID: NCT00327509). All recruited persons were informed about the study and consented the data use.

### Isolation of mononuclear blood cells

Individual blood samples (20 ml) anti-coagulated with heparin were collected from patients and controls. Leukocytes were separated using Ficoll-Histopaque gradients (Histopaque 1077; Sigma Aldrich, St. Louis, MO) as described previously [9]. After washing procedure (three times) with physiological buffer solution (PBS, Biochrom AG, Berlin, Germany) cells were pelleted and immediately frozen on dry ice and stored at -80 °C till molecular biological analysis.

### Isolation of total RNA, mRNA, and first-strand cDNA synthesis

Isolation of total RNA from aliquoted samples of mononuclear blood cells were performed using Total RNA Isolation Reagent (Thermo Fisher Scientific, ABgene product line, Epsom, UK). After storing for 5 min on ice, 0.2 ml chloroform per 1 ml RNA reagent was added. The samples were vortexed and placed on ice for 5 min. The homogenate was centrifuged at 12,000× g (4 °C) for 15 min. RNA samples were transferred into new tubes. Equal volume of isopropanol was added. Samples were stored overnight at -80 °C. After a 30 min centrifugation at 12,000× g (4 °C) total RNA was sedimented. RNA samples were washed twice with 70% ethanol and briefly dried. Total RNA Pellets were dissolved in diethylpyrocarbonate (DEPC)-water. The isolation of mRNA was performed with an Oligotex mRNA Mini Kit (Qiagen, Hilden, Germany). Total RNA was mixed with Oligotex Suspension consisting of polystyrene-latex particles linkaged with dC_10_T_30_ oligonucleotides. mRNA samples were hybridized to Oligotex. mRNA-Oligotex complexes were loaded on spin column, washed three times. mRNA were eluted from column with sterile water. cDNA synthesis was performed using the iScript^TM^ cDNA Synthesis Kit (Bio-Rad, Hercules, CA). The reaction mix (mRNA Template, 5× iScript reaction mix and iScript reverse transcriptase) was incubate for 5 min at 25 °C, 30 min at 42 °C and 5 min at 85 °C. The synthesized cDNA was stored at -20 °C till molecular biological analysis.

### Hybridization to Atlas Human^TM^ Cardiovascular Array

#### Preparation of biotin-labeled cDNA probes

cDNA (100 ng) was labeled using a SpotLight^TM^ Random Primer Labeling kit (Clontech, Mountain View, CA). Template cDNA together with 5 µl of 10× Random Primer mix were heated to 97 °C for 3 min in a final volume of 31 µl and chilled quickly on ice. After adding the reaction mix (5 µl 10× Klenow reaction buffer, 5 µl 10× Klenow labeling mix, 1 µl Klenow enzyme, and 8 µl sterile water [Clontech]), the labeling reaction was performed at 37 °C for 30 min. The reaction was then stopped by adding 2 µl of 0.5 M EDTA (pH 8.0).

#### Purification of biotin-labeled probe

Unincorporated biotin-labeled nucleotides and small (<0.1 kb) cDNA fragments were removed using the NucleoSpin extraction columns (Clontech). The labeled probe was mixed with binding buffer and loaded directly on spin column. After centrifugation the column was washed three times and dried briefly. Elution buffer was loaded onto the filter. Biotinylated probe was collected after brief centrifugation. The concentration of the newly synthesized biotin-labeled probes was determined by UV spectroscopy. Finally, the biotin-labeled probes were stored at –20 °C until hybridization to the Atlas™ Array (Clontech).

#### Hybridization of biotin-labeled probes to Atlas Array

For the hybridization, Atlas^TM^ Human Cardiovascular Array (Clontech) and SpotLight^TM^ Chemiluminescent Hybridization and Detection kit (Clontech) were used. Each Atlas array (membrane) was wetted by placing it in a dish of de-ionized H_2_O, after which it was transferred to the hybridization bottle and pre-hybridized in the hybridization mix at 42 °C for 3 h before the hybridization. Each hybridization reaction was performed overnight at 42 °C with individual biotinylated cDNA probes. These probes were denatured in 100 mM NaOH at 68 °C for 20 min and neutralized with 0.5 M NaH_2_PO_4_ (pH 7.0) at 68 °C for 10 min before the overnight hybridization in the Hybridizer (Techne Inc., Burlington, NJ).

#### Stringency washes

The hybridization solution was discarded and the membranes were washed four times in 200 ml of wash solution 1 (2× saline-sodium citrate [SSC] buffer and sodium dodecyl sulfate [SDS]) for 30 min at 60 °C with continuous agitation. Wash solution 1 was then replaced with wash solution 2 (0.1× SSC and 0.5% SDS), and the membranes were washed two more times for 30 min at 48 °C.

#### Probe detection and signal visualization

The membranes were incubated first in 25 ml blocking buffer per Atlas array at room temperature for 1 h before the incubation with streptavidin-horse-radish peroxidase conjugate (final dilution of 1:300) for 1 h with constant gentle agitation. Afterwards the membranes were washed four times in 1× wash buffer (SpotLight^TM^ Chemiluminescent Hybridization and Detection kit; Clontech) for 10 min with concomitant equilibration in substrate equilibration buffer for 5 min at room temperature before the incubation in 8 ml working solution (the mix of luminol/enhancer solution and stable peroxide solution) per membrane for 5 min. After removing excess liquid, the Atlas arrays were processed for autoradiography with exposure times of 1, 2, 5, 10, and 30 min. The exposed spots were further scanned and analyzed with AtlasImage^™^ 2.0 software (Clontech).

### Reverse transcriptase PCR and real-time quantitative PCR 

In order to detect the expression of the target genes in leukocytes and to optimize the reaction conditions for real-time quantitative PCR, reverse transcriptase PCR (RT-PCR) was performed using specific primer sets (Table 1). cDNA synthesis was performed using the iScript^TM^ cDNA Synthesis kit (Bio-Rad). The PCR mixture contained 1× PCR buffer (16.6 mM ammonium sulfate, 67 mM Tris, pH 8.8, 6.7 mM MgCl_2_, 10 mM 2-mercaptoethanol), dNTPs (each at 1.25 mM), primer pairs (100 pM each per reaction), and 10 ng of cDNA template in a final volume of 50 µl. Reactions were hot-started at 95 °C for 5 min before adding 1.5 units of Taq polymerase (Red-Hot^®^, Thermo Fisher Scientific, ABgene product line) at the annealing temperature of 56 °C, followed by polymerization at 72 °C for 1 min. Amplification was carried out in DNA Thermal Cycler TC480 (Perkin Elmer, Waltham, MA) for 45 cycles (denaturation for 45 s at 95 °C, annealing for 45 s at 56 °C, and polymerization at 72 °C for 30 s), followed by a final 7-min extension at 72 °C. Negative controls without DNA as well as positive controls with a sequenced template were performed for each set of PCR experiments. PCR products (50 µl) were directly loaded onto 3% agarose gels (wide range-agarose gels for analysis of DNA fragments longer than 50 bp; Sigma), stained with ethidium bromide after electrophoresis, directly visualized under UV illumination, and imaged using a specialized imaging system (Eurofins MWG Operon, Ebersberg, Germany). The specificity of each PCR amplification was controlled using the site-specific restriction analysis of target PCR products. The amplification products underwent an extraction from the agarose gel, using a DNA isolation kit (DNA Gel Extraction Kit; Fermentas, Vilnius, Lithuania) before digestion. The products were digested in a final volume of 50 µl with 20 units of each restriction endonuclease (see Table 1) for 2 h, according to conditions specified by the manufacturer (Fermentas), and imaged after electrophoresis.

**Table 1:** Primer sets and corresponding lengths of the expected PCR products.

**Genes**	**Forward Primer**	**Reverse Primer**	**Product size, bp**	**Endonucleases for product digestion**
*β-Actin*	TAAGGAGAAGCTGTGCTACG	TGAAGGTAGTTTCGTGGATG	203	GsuI, LguI (SapI)
*P2Y*	TCATCTCTCTGCTGGCTATC	CTTGGTGCGTAGCTTCTG	347	RsaI, XbaI
*ICAM1*	AGTCACCTATGGCAACGAC	GCCTCACACTTCACTGTCA	216	AluI, PstI
*Na+/Ca2+ EP1*	ACCACCAAGACAACTGTGAG	CACGCAAATGCTTAATCTTC	244	CfrI (EaeI), PstI
*MT1-MMP*	ATCTGTGACGGGAACTTTGA	CTTCCTCTCGTAGGCAGTG	174	HhaI, HphI

In order to profile precisely the changes in an expression of target genes, real-time quantitative PCR was used. SYBR^®^ Green I (Invitrogen, Molecular Probes^®^, Carlsbad, CA) was used as the intercalation dye and fluorescent reporter molecule detecting the accumulation of the amplified double-stranded product in the iCycler iQIM Detection System (Bio-Rad). The synthesized cDNAs (50 ng; see RT-PCR) were used for each real-time PCR analysis. The reaction mixtures had the same contents as for RT-PCR with the exception of Red-Hot^®^ polymerase (Thermo Fisher Scientific, ABgene product line), which was substituted for Thermoprime Plus DNA polymerase (Thermo Fisher Scientific, ABgene product line) in order to avoid color signal disturbances. The same amplification program was used in both qualitative RT-PCR and quantitative real-time PCR analysis. The algorithm of the iCycler iQIM detection system normalizes the reporter signal (non-intercalated SYBR^®^ Green I) to a passive reference and multiplies the standard deviation (SD) of the background signal in the first few cycles by a default factor of 10 to determine a threshold. The cycle at which this baseline level is exceeded is defined as the threshold cycle (C_t_). C_t_ depends on the initial template copy number and is proportional to the log of the starting amount of nucleic acid [11]. The data are normalized by subtracting the difference of the C_t_ values of a target gene from those of the housekeeping one (β-actin). The relative expression levels were calculated for each sample based on the differences in C_t_ values [11].

### Statistical evaluation

Statistical significance was calculated by the two-sided unpaired Student *t* test and was considered significant at the p<0.05 level.

## Results

### Expression array

**Figure 1:**
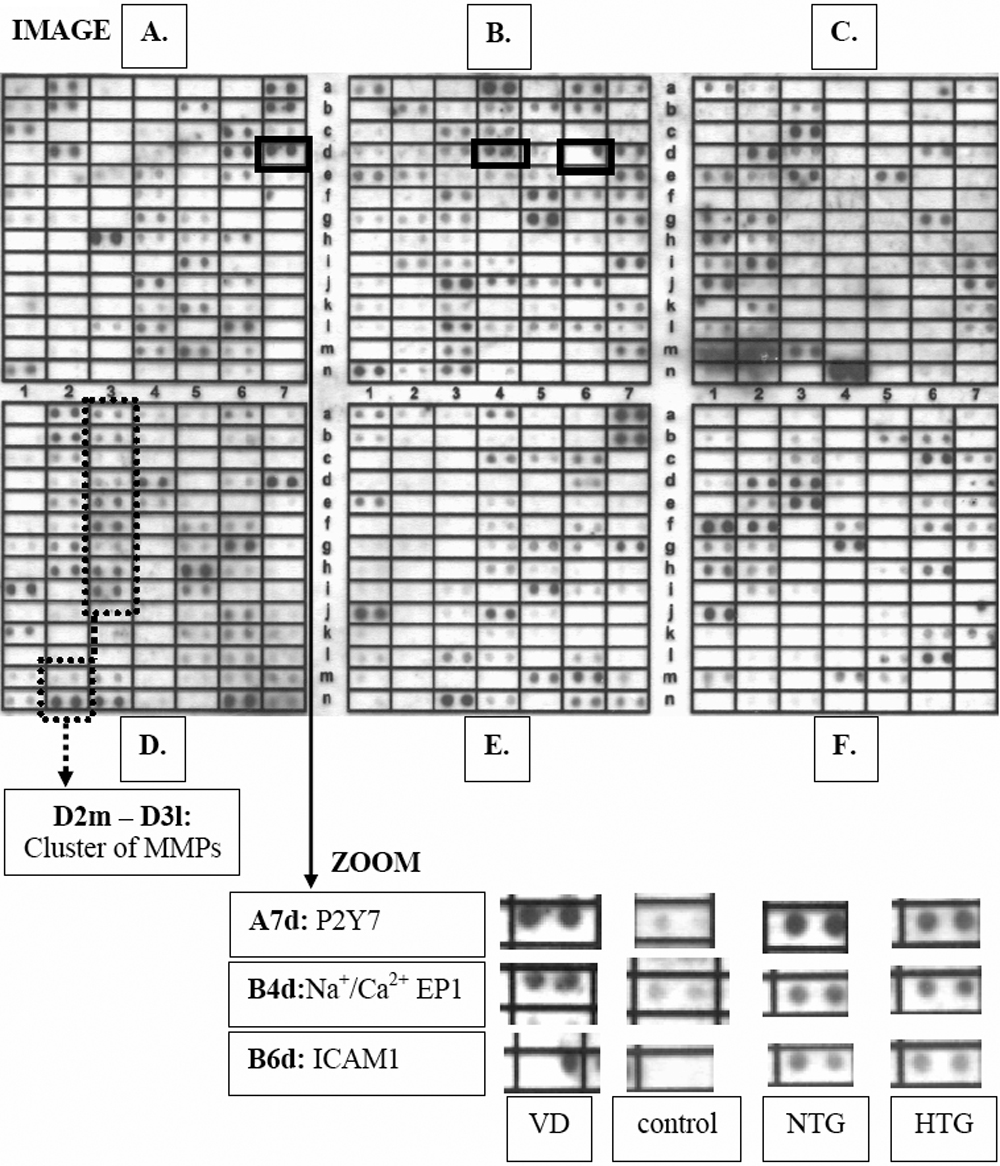
Expression Array image. The image of 588 genes (see Atlas^TM^ Human Cardiovascular Array in Methods) is shown specifically for the group of individuals with vascular deregulation. The double spots marked with black frames show the most stable expressional difference in vascular deregulation (VD), normal-tension (NTG) and high-tension (HTG) glaucoma patients groups versus controls. These double-spots are zoomed for the groups tested (ZOOM), and the names of corresponding genes as well as their positions in Atlas are given.

The image of hybridized Atlas™ Human Cardiovascular Array is represented in Figure 1. The similarities as well as alterations in gene expression among NTG, HTG, and VD groups versus controls are summarized in Table 2.

**Table 2:** Numbers of genes an expression of which is either differential or equal among the groups tested as shown by “Expression array”.

**Differential to control**	**VD equal to**	**VD Differential to**
NTG→146	Control →528	NTG→109
HTG →68	NTG→48	K→60
VD→60	HTG→43	HTG→43
NTG = HTG→53	**NTG = HTG →34**	NTG = HTG→21

Thereby, 108 genes were found to be differentially expressed between NTG and HTG groups. 34 genes demonstrated similar alteration for vasospastic individuals (VD) and both glaucoma-patient groups when compared to the healthy controls (see these genes listed in Table 3).

Compared to the control group, the expression of 146, 68, and 60 genes was found to be altered in NTG, HTG, and VD groups, respectively; the same 53 genes were differentially expressed in both NTG and HTG groups versus controls. Among 146 genes differentially expressed specifically in the NTG group, we monitored 48 and 53 genes that were similarly expressed either in VD or in HTG groups, respectively. Among 68 genes differentially expressed specifically in the HTG group, we found 43 genes to be similarly expressed in the VD group only. The highest difference—146 genes—was found to be between the NTG and control groups. In contrast, the lowest difference—21 genes—was demonstrated to be between VD and the overlap of NTG/HTG.

Thirty-four genes demonstrated similar expressional alterations in NTG, HTG, and VD groups versus controls, as presented in Table 3. As the differentially expressed overlap VD/NTG/HTG was compared with the control group, the following most significant differences were monitored:

**Table 3:** Differentially expressed genes versus controls.

**Double-spot position in “EA”-image exp. difference versus control**	**Name of gene as given in “AtlasTM Human Cardiovascular Array”**	**GenBank accession**	**SwissProt accession**	**Gene/Protein classification**
**BLOCK A**				
A7d increased	P2Y purinoceptor 7 (P2Y7); leukotriene B4 receptor; Chemoattractant receptor-like1 (CMKRL1)	U41070	Q15722 ; Q13305; Q92641	Other Receptors (by Ligands) G Protein-Coupled Receptors
A7e increased	Retinoic acid receptor gamma 1 (RAR-gamma 1; RARG)	M24857 ; M38258; M57707; M32074	P13631	Transcription Activator & Repressors; Hormone Receptors; Nuclear Receptors
**BLOCK B**				
B1n increased	Androgen receptor coactivator 70-kDa subunit (ARA70)	L49399	Q13772	Transcription Activator & Repressors
B4c increased	G protein-activated inward potassium channel 4 (GIRK4); heart K+/ATP channel (KATP1); cardiac inward rectifyer (CIR); KIR3.4	U39195	P48544 ; Q92807	Voltage-gated Ion Channels
B4d increased	Sodium/calcium exchanger 1 precursor; Na+/Ca2+-exchange protein 1	M91368	P32418	Symporters & Antiporters
B4e increased	Multidrug resistance protein 3 (MDR3); P-Glycoprotein 3 (PGY3)	M23234	P21439	Drug-Resistance Proteins; Xenobic Transporters
B5f increased	Endothelial nitric oxide synthase (EC-NOS)	M93718	P29474	Other Metabolism Enzymes; Other Intracellular Transducers, Effectors & Modulators
B6d increased	Intercellular adhesion molecule 1 precursor (ICAM1); major group rhinovirus receptor; CD54 antigen	J03132	P05362	Matrix Adhesion Receptors
7g increased	Calcium & integrin-binding protein (CIB)	U85611	Q99828	Calcium-Binding Proteins
1g increased	Cadherin 7 (CDH7)	AF047826	O60574	Cell Surface Antigens; Cell-Cell Adhesion Receptors
1h increased	Intestinal peptide-associated transporter 1 (HPT1)	U07969	Q12864	Other Cell Adhesion proteins; Other Cell Adhesion Proteins; Other Facilitated Diffusion proteins
2i increased	GAP junction alpha-5 protein	L34954	P36382	Cell-Cell Adhesion Receptors; Other Membrane Channels & Transporters
3m increased	Integrin beta 2 (ITGB2); cell surface adhesion glycoprotiens LFA-1/CR3/p150, 95 beta subunit precursor; CD18 antigen; Complement receptor C3 beta subunit	M15395	P05107 ; Q16418	Cell-Cell Adhesion Receptors
1m increased	Cardiac LIM domain protein; muscle LIM protein; cystein-rich protein 3 (CRP3); LIM-only protein 4	U49837	P50461	Basic Transcription Factors; Other Transcription Proteins; DNA Synthesis, Recombination & Repair Proteins
1n increased	Cardiotrophin-1 (CT1)	U43030	Q16619	Growth Factors, Cytokines & Chemokines
2n increased	Matrix metalloproteinase 16 (MMP-16)	D83646	P51512	Chromatin Proteins; Metalloproteinases
4a increased	TIMP-3	U14394	P35625	Extracellular Matrix Proteins; Proteinase Inhibitor
4b increased	TIMP-4	U76456	Q99727	Extracellular Matrix Proteins; Proteinase Inhibitor
4d increased	Sterol regulatory element-binding transcription factor 1	U00968	P36956	Basic Transcription Factors; Other Apoptosis-Associated Proteins
4e increased	Sterol regulatory element-binding transcription factor 2	U02031	Q12772	Basic Transcription Factors; Other Apoptosis-Associated Proteins
5h increased	Rab geranylgeranyl transferase bety subunit	Y08201	P53611 ; Q92697	Trafficking & Targeting Proteins; Protein Modification Enzymes; GTP/GDP Exchangers & GTPase Activity Modulators
6m decreased	Muscle-specific Dnase I-like precursor (Dnase 1L1; DNL 1L); Dnase X	X90392 ; L40817; U06846	P49184	DNA Synthesis, Recombination & Repair Proteins; Apoptosis-Associated Proteins
1b increased	Lanosterol synthase (LSS); oxidosqualene lanosterol cyclase (OSC)	D63807	P48449	Complex Lipid Metabolism
3n increased	NADPH-cytochrome p450 reductase	S90469	Q16455 ; P16435	Xenobic Metabolism
4g increased	Steroid 5 alpha reductase 1 (SRD5A1); 3-oxo-5-alpha steroid 4 dehydrogenase 1	M32313 ; M68886	P18405	Complex Lipid Metabolism
4h increased	Steroid 5-alpha reductase 2 (SRD5A2); 3-oxo-5-alpha steroid 4 dehydrogenase 2	M74047	P31213	Complex Lipid Metabolism
2d increased	Pregnane X receptor (PXR)	AF061056	O75469	Hormone Receptors; Nuclear Receptors
F2e increased	Estrogen-related receptor gamma	AF058291	O75454	Hormone Receptors; Nuclear Receptors
F2f increased	Nuclear receptor subfamily 4 group A member 2 (NR4A2); nuclear receptor-related protein 1 (NURR1); transcriptionally inducuble nuclear receptor (TINUR); NOT	X75918	P43354	Hormone Receptors; Nuclear Receptors; Transcription Activators & Repressors
2i increased	Orphan nuclear receptor TR4; nuclear receptor subfamily 2 group c member 2 (NR2C2); TAK1	U10990	P55092 ; P49116	Orphan Receptors; Nuclear Receptors; Transcription Activators & Repressors
3a increased	RAR-related orphan receptor C	U16997	P51449	Orphan Receptors; Nuclear Receptors; Transcription Activators & Repressors
3e increased	LX receptor alpha (LXR alpha)	U22662	Q13133	Orphan Receptors
3i increased	Platelet-activating factor acetylhydrolase IB alpha subunit	L13387	P43034	Other Metabolism Enzymes
7c increased	Myocyte-specific enhancer factor 2A (MEF2A); serum response factor-like protein 1	X68505	Q02078 ; Q14223; Q14224	Basic Transcription Factors

Differentially expressed genes (altogether 34 ones as also summarized in Table 1) versus controls, the transcriptional levels of which were similar for VD, NTG, and HTG groups; the most significant differences compared to controls are given in thick letter. “Expression Array” (“EA”)-image is given in Figure 1; “exp. difference” means an alteration in transcriptional level compared to that of control.

- P2Y purinoreceptor 7
- Na^+^/Ca^2+^ exchange protein 1 (Na^+^/Ca^2+^ EP1)
- intercellular adhesion molecule 1 (ICAM1)
- cluster of tissue-remodeling metalloproteinases.

The corresponding images for the groups tested are shown in Figure 1.

### Real-time PCR

**Figure 2:**
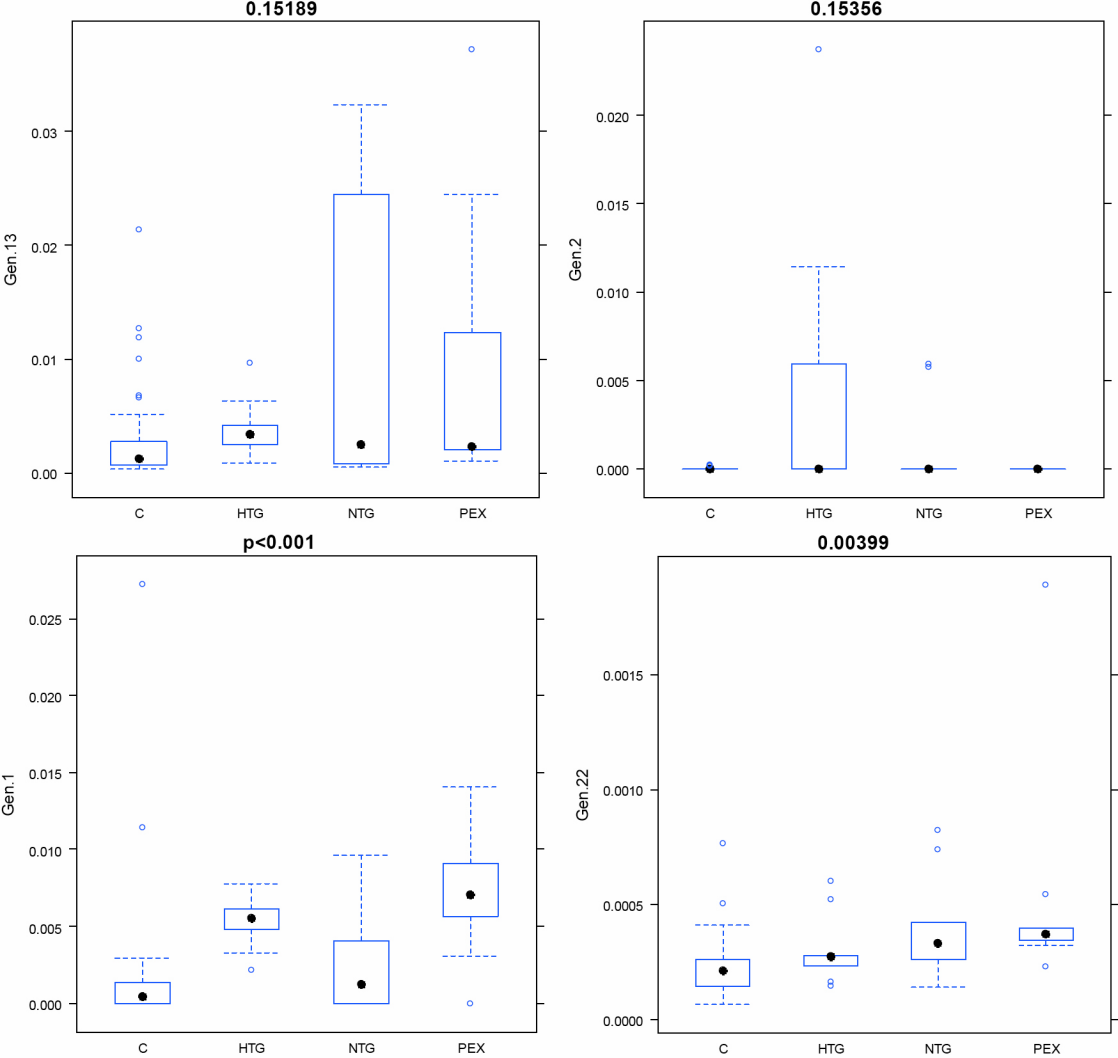
Comparative real-time PCR. The measurements for the most stable alterations (see the “Expression array” images as given in Figure 1) were performed in glaucoma patients compared to controls. All measurements have been performed by a blind study. The names of the encrypted genes are following: the gene 1=P2Y (purinoceptor 7); the gene 2=ICAM 1 (intercellular adhesion molecule 1); the gene 13=Na^+^/Ca^2+^ EP1 (Na^+^/Ca^2+^ exchange protein 1); the gene 22=MT1-MMP (membrane type 1 matrix metalloproteinase).

The most stable, i.e., statistically significant alterations in target transcripts for glaucoma patients versus controls are shown in Figure 2.

#### P2Y expression values (encrypted gene 1)

The most significant alteration in the transcription regulation of this gene for glaucoma patients has been demonstrated. If any, only traces of this transcript were detected in the control blood samples. In contrast, significantly increased expression rates were demonstrated for glaucoma patients.

#### ICAM1 expression values (encrypted gene 2)

Traces of this transcript were found in a few control blood samples. In contrast, some of the NTG and HTG patients demonstrated highly increased expression levels.

#### Na^+^/Ca^2+^ EP1 expression values (encrypted gene 13)

An upregulation of this transcript has been demonstrated for glaucoma patients compared to controls.

#### Membrane type 1 matrix metalloproteinase (MT1-MMP) expression values (encrypted gene 22)

A significant upregulation of this transcript has been demonstrated for glaucoma patients compared to controls.

## Discussion

### Why is early diagnostics important for glaucoma treatment?

Worldwide, 67 million people are affected by the neurodegenerative eye disease glaucoma. GON is the second leading cause of permanent vision loss; it is a chronic degenerative process, the onset of which is not possible to monitor by currently existing diagnostic tools. Early treatment has been demonstrated to be highly beneficial for well-timed treatment measures to slow disease progression [12].

### What is the potential impact of predictive molecular diagnostics in glaucoma?

Molecular pathomechanisms of glaucoma demonstrate both a considerable overlap and remarkable particularities to some other neurodegenerative disorders, such as Alzheimer’s and Parkinson’s diseases [13]. Thus compared to controls the NTP demonstrates enhanced expression levels in glaucoma, patients with Down syndrome, Alzheimer’s disease, and some other neurodegenerative diseases indicating axonal lesions. However whereas the accumulation of the TAU protein is characteristic for Alzheimer’s disease, glaucoma patients do not demonstrate an increase in the target protein versus controls [12]. Therefore, monitoring of the pathology-specific molecular patterns is particularly valuable for the development of reliable diagnostic approaches before the manifestation of the pathology.

### What is the impact of vascular deregulation in glaucoma pathology?

A wealth of literature points to the importance of hemodynamics in glaucoma pathology. Vasospasm, defined as an inappropriate constriction or insufficient dilatation in the microcirculation, is frequently observed in glaucoma patients. Moreover, this is also a frequent phenomenon in young female individuals, which makes the task of glaucoma prediction and targeted prevention particularly attractive from several points of view.

### Similarities in subcellular images detected in circulating leukocytes of vasospastic individuals and glaucoma patients.

DNA breaks have been been analyzed by the Comet Assay technology, and DNA damage has been monitored in circulating leukocytes of both glaucoma patients and vasospastic individuals compared to healthy controls. Further, quantitative Comet Assay analysis demonstrated significantly enhanced levels of DNA breaks in glaucoma patients compared to both healthy vasospastic and non-vasospastic individuals and revealed pathology-specific comet patterns [14]. From this viewpoint, vasospasm can be considered as risk factor for glaucoma, and Comet Assay imaging technology is a promising approach to be applied for early/predictive diagnostics.

### Similarities in expression patterns detected in circulating leukocytes of vasospastic individuals and glaucoma patients.

**Figure 3:**
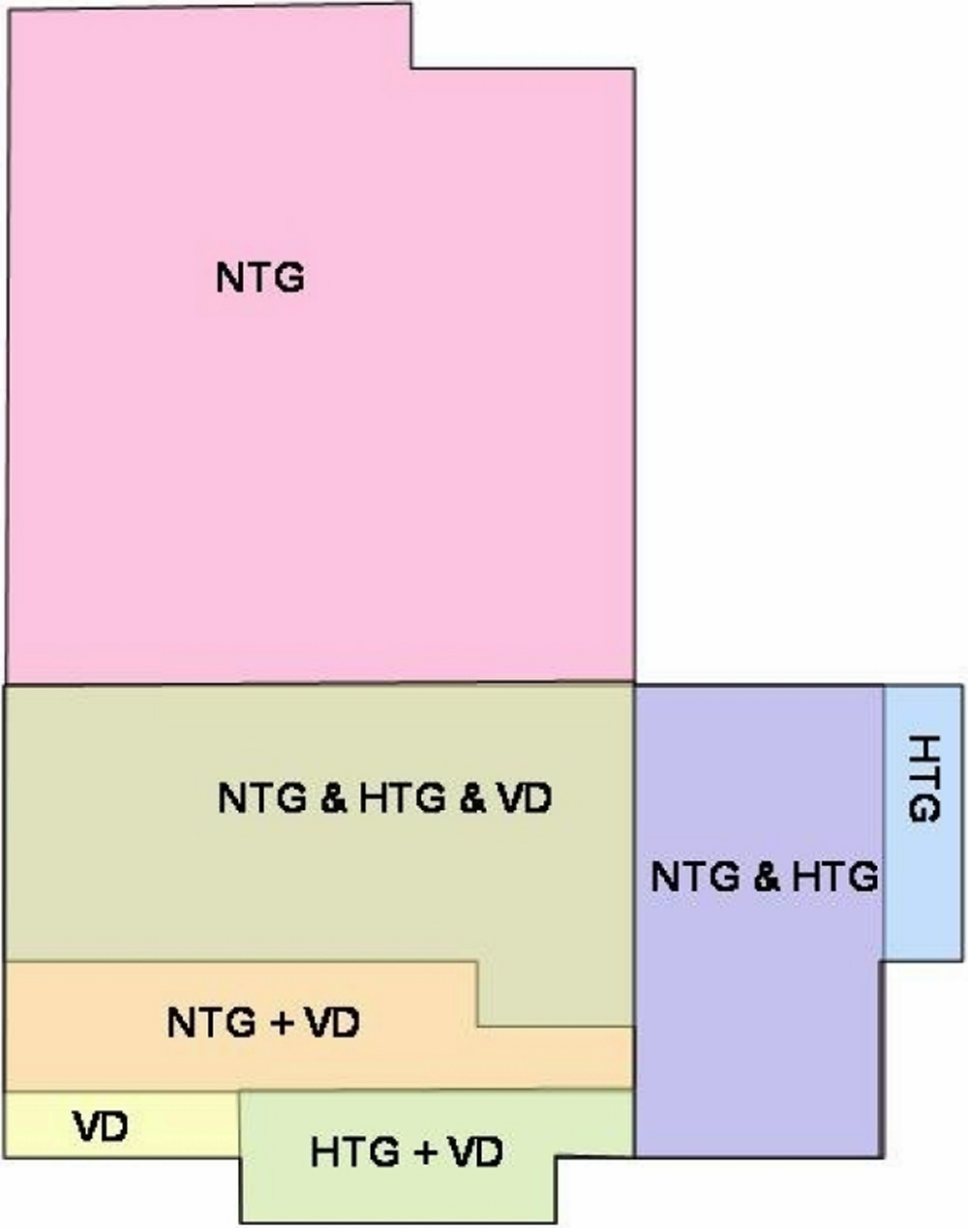
Overview of protein expression profiling. The diagram demonstrates the overlap in molecular mechanisms among single pathologies according to the similarities in gene expression patterns in vascular deregulation (VD), normal-tension (NTG) and high-tension (HTG) glaucoma patients groups. Single disorders demonstrate some pathology-characteristic expressions marked by the corresponding symbol, e.g., NTG. Further, there are partial expression overlaps between two of three pathologies symbolized by the corresponding pair, e.g., NTG & HTG. The similarities among all three groups (NTG & HTG & VD) of comparison are considered as the central issue that this study is focused on. Altogether 34 genes that create this overlap are summarized in Table 3, providing the clue to the molecular pathomechanisms that potentially predispose vasospastic individuals to the glaucomatous pathology.

The following key pathways are affected in glaucoma pathology: stress response, apoptosis and DNA-repair, adhesion, blood-brain-barrier breakdown, tissue remodeling, transcription regulation, multidrug resistance, and energy metabolism [13]. Here we demonstrate significant similarities in expression patterns of vasospastic individuals and glaucoma patients as compared to healthy controls (see Figure 3).

#### P2Y purinoreceptor is upregulated in vasospastic individuals and glaucoma patients

The movement of leukocytes from blood into tissue is regulated by local production of chemoattractants—diverse molecules, the chemotactic signal of which is transmitted by G-protein-coupled purinoceptor family P2Y. These receptors respond to chemotactic signals of traumatic, infectious, post-ischemic, autoimmune, and various toxic injuries. Extracellular nucleotides released from the activated platelets and other damaged cell types exacerbate the inflammatory response by cell-specific leukotrene generation [15]. Thus, neutrophils generate leukotrienes B4 (LTB4), which are involved in the genesis of inflammation and edema because of their effect on vascular permeability, plasma extravasation, diapedesis of white blood cells, and their important role in an adaptive immune responses, as reviewed by Di Gennaro et al. [16]. Specifically, a highly enhanced concentration of leukotrienes B4 and C4 has been observed in cerebrospinal fluid of patients with multiple sclerosis [17]. This gene’s product is the member of the leukotriene receptor family (LTB4 receptor or P2Y purinceptor 7), and for the first time, has been isolated from the human erythroleukemia cell cDNA library [18]. One of the physiological roles of LTB4 is the stimulation of monocytes, neutrophils and endothelial cells [19]. There is a growing body of evidence indicating an important role of LTB4 receptors in the regulation of pathologic inflammation. Particularly using animal inflammatory models, a reduced disease severity has been shown when LTB4 receptor antagonists have been applied; the same effect has been observed in mice with target deletion of BLT1—a high-affinity LTB4 receptor primarily expressed in leukocytes [20]. Furthermore, some studies support a potential role of P2Y receptors in controlling IOP, although additional investigations of this issue are necessary [21].

#### ICAM-1 is upregulated in vasospastic individuals and glaucoma patients

Neutrophil–endothelium interactions are implicated in pathological alterations of blood vessel function, potentially leading to circulatory disturbances [22]. Interactions between blood cells and the vessel wall result in endothelial dysfunction and injury leading to increased blood–brain barrier permeability and even edema formation [23]. Penetration of leukocytes into inflamed areas involves a complex interaction of leukocytes with endothelium through regulated expression of surface adhesion molecules. Found in this work to be highly expressed in VD, NTG, and HTG groups, ICAM-1 is believed to be largely responsible for the adhesion and transendothelial migration of leukocytes [24]. This is in agreement with earlier developed strategies aimed at inhibition of endothelial interactions with leukocytes via use of adhesion molecule monoclonal antibodies, which successfully reduce cerebral ischemia/reperfusion injury, infarct size, and demonstrate a neuroprotective effect generally [25–27]. In our study, highly expressed ICAM-1 was found in leukocytes of glaucoma patients; in contrast, only traces, if any, of the target expression was detected in the leukocytes of healthy controls.

#### Sodium calcium exchanger

Many studies have examined the levels of cytosolic Ca^2+^ [Ca^2+^]_c_ and Na^+^ [Na^+^]_c_ in human blood cells. Leukocytes have been the main target when studying the relationship between blood pressure and intracellular content of both ions, as reviewed by Horiguchi et al. [28]. As shown by Horiguchi et al. [28], the resting [Ca^2+^]_c_ correlates well with sodium calcium exchanger (NCE) expression, indicating NCE expression regulation to be an adaptive mechanism for Ca^2+^ extrusion mediation. Horiguchi et al. also observed a gender effect on [Ca^2+^]_c_ / [Na^+^]_c_ regulation in circulating leukocytes being in relationship with blood pressure. Further, the role of endothelial intracellular Ca^2+^ concentration in molecular mechanisms of vasoconstriction/vasodilatation has been intensively studied, and the functional association between P2Y purinoceptors, endothelial nitric oxide synthesis, and calcium transport in terms of vascular regulation is well documented in the literature [29,30]. Our findings here clearly demonstrate the upregulation of both P2Y purinoceptor and Na^+^/Ca^2+^ exchanger in circulating leukocytes of glaucoma patients as well as vasospastic individuals versus healthy controls.

### Concluding remarks

We conclude that

- The expressional overlap between NTG and HTG groups versus controls (here 53 genes) indicates broad similarities in pathomechanisms of both glaucoma forms.
- The expression similarities (altogether 34 genes) found here between glaucoma and VD versus controls indicate, on one hand, a predisposition of VD individuals to glaucomatous damage, and, on the other hand, an important role of the vascular component in the pathogenesis of glaucoma.
- Expression differences (altogether 21genes) between VD and glaucoma patients might indicate some glaucoma-specific pathomechanisms.
- Both groups of genes (34 and 21) could be potentially useful for glaucoma diagnostics.

As we show here, this molecular rearrangement in leukocytes of both VD and glaucoma patients includes an upregulated adhesive protein expression via ICAM1; an induced chemotaxis via P2Y purinoceptors; a mobilization of intracellular Ca^2+^ response via Na^+^/Ca^2+^ exchanger; and a core of tissue-remodeling metalloproteinases.

This molecular rearrangement has been shown to be typical for circulating leukocytes during vascular injury, as reviewed by Kunapoli and Daniel [31]. These pathology-specific molecular patterns in blood may create the basis for the development of novel noninvasive molecular imaging technologies in early and predictive glaucoma diagnostics.
